# “Don’t let me be misunderstood”: communication with patients from a different cultural background

**DOI:** 10.1007/s00467-022-05573-7

**Published:** 2022-08-05

**Authors:** Christina Taylan, Lutz T. Weber

**Affiliations:** Pediatric Nephrology, Children’s and Adolescents’ Hospital, University Hospital of Cologne, Faculty of Medicine, University of Cologne, Kerpener Str. 62, 50937 Cologne, Germany

**Keywords:** Cross-cultural patient care, Immigrants, Ethics, Cultural competence, Communication skills

## Abstract

In recent years, migration and the social changes associated with it have increasingly become the focus of scientific interest. The diversity of cultures in hospitals poses a major challenge. Medical teams are often confronted with language barriers and different concepts of illness, health, and healing. The field is wide, and in addition to foreign language skills, primarily human skills such as self-awareness, communication, and empathy are demanded. Religion also plays a role in medical care for patients with a foreign cultural background. This work is intended to provide an overview of the scientifically based necessary skills in dealing with this patient clientele and to give an insight into the personal experiences of the authors. After many years of dealing with intercultural care of patients, this experience has shown one thing above all: Sometimes, it is beyond language and just needs humanity.

## Background

Migration is an important issue that affects all areas of public life. Almost 272 million people, or about 3% of the world’s population, live outside the country in which they were born. As of January 1, 2020, almost 37 million of the European Union’s 447.3 million residents were born outside the EU [1]. Proportionally, 21% of the residents in Europe and 13% in the USA are migrants or have a migration background [2]. Among other things, this fact entails the need to provide healthcare and education to meet the needs of daily life for these people in their new home. Culture and ethnicity can cause difficulties in establishing a sustainable doctor–patient relationship. Literature shows that insufficient language skills and cultural barriers negatively affect care for migrants resulting in reduced access, higher hospitalization rates, increased risk of permanent damage, and limited health knowledge [3–5]. Health-related quality of life has been shown to be worse in children of non-Western origin with kidney failure, despite living in Western European countries [6]. A multicultural society places new demands on healthcare workers, especially in the hospital, where care is particularly personal and unavoidable. The limited resources available in terms of personnel, training budgets, and time, as well as language barriers on both sides, make it difficult to provide optimal intercultural patient care. Cultural differences in the understanding and acceptance of illness, traditions, and different expectations of the healthcare system also make it difficult to treat these patients well [4, 7, 8]. Intercultural communication is crucial in this context and is becoming increasingly important. To communicate efficiently with patients of foreign origin is a major task today [9].

While there is a lot of literature on intercultural communication from the field of business management and at least some literature from nursing, there is very little data on intercultural communication from the pediatric medical profession. The topic is enormously comprehensive and touches language, culture, faith, and general ethics. While this review cannot fully cover all these aspects, it may encourage the reader to confront this sensitive topic, and to identify one’s own strengths and limitations.

## Communication

The aim of communication is to share and understand a meaning or to “make things common” [10]. In a doctor-patient/parent relationship, communication must lead to the successful transmission of information concerning a diagnosis or a treatment. Good communication, respecting the sender’s and recipient’s perspective, is demanding. This is especially true in emotional situations that are the rule in healthcare. The physician would probably assess the communication successful when all information has been given with empathy. The patient/parent would probably assess the communication successful when they feel well informed, have been allowed to ask all their questions, and get the feeling of receiving patient-centered care. There are no general standards of good communication. Communication is influenced by emotions, education, situations, circumstances, attitudes, habits, etc. and is hence highly variable. Communication styles differ between occupational groups, social groups, age groups, and among members of a family, and, of course, they differ between cultures.

In some cultures, senders are used to being open, frank, and honest when giving their opinion. If they say something is “fine,” then it is just fine. Some cultures are very different. One needs to sense much more than words express. People can say one thing but mean another. Saying something is “fine” sometimes means it is not fine, possibly it is the exact opposite. Those who have been socialized in the same way can understand in what sense a word was used. Cultural differences clearly shape language style. Some value transparency whereas others value diplomacy, some value hierarchy whereas others value equality, some value reputation and formality, whereas others value humor, friendship, and a much more informal approach to communication.

## Culture

The first years of life are marked by observational learning, but the subsequent cultural rules of a society are primarily learned through communication. Culture also influences communication beyond language. The way of formulating a request or asking for directions is strongly influenced by culture. Perception, thinking, and acting are guided by cultural affiliation. Assumptions, values, and behavior patterns can lead to mistaken assignments of meaning and misinterpretations. Misunderstandings also occur in an intercultural communication between two obviously similar cultures, due to a false assumption of a common background. “Cultural competence” is generally defined as what one can learn from a particular group of relevant attitudes, values, beliefs, and behaviors as a non-member of that culture. It is often subdivided according to ethnicity, race, religion, or into national groups such as “Europeans,” “African Americans,” “Hispanics,” or “Asians.” This disregards the fact that culture is multidimensional and dynamic [11]. Some people in an ethnic group may have a different understanding of disease and its condition (their “model of explanation”) than others in the same group. It is important to keep in mind that culture is not just a single aspect, but encompasses multiple aspects, all of which influence behavior [12]. Cultural processes often differ within the same ethnic or social group because of differences in age cohort, gender, political association, religion, ethnicity, and even personality [12]. Similarities in physical appearance and racial or ethnic affiliation must not lead to the assumption that these people have the same beliefs, values, and behaviors, i.e., that they automatically share “cultural concepts” [13]. This approach can result in stereotypical thinking rather than cultural competence. International patient care should therefore not be reduced to “cultural categories,” but instead competencies should focus on foundational communication skills.

The communication skills noted above should be applied to patient-centered communication (PCC), a term that has been used to describe a group of communication strategies and behaviors that promote reciprocity, shared understanding, and shared decision-making in healthcare. A patient-centered conversational approach can help identify problems and health-related perceptions in all patients, regardless of culture [14]. With this form of interviewing, the patient must be perceived at all levels. Different styles of communication and decision-making are considered, as well as the patient’s role within the family, and their mistrust and prejudices. In study from the USA, Street et al. analyzed the different influences on the communication and perception of physicians. The patient’s communication style had the greatest influence on a doctor’s communication behavior. Physicians were more patient-centered in patients who were active and engaging during a conversation, and then showed more positive feelings when patients also showed positive feelings. Conversely, they were more easily irritable in irritated patients. The authors concluded that the mutual influence of physician and patient has a strong influence on the interaction, as both positive and negative perceptions could be transmitted in both directions from one interlocutor to another [15].

The best way to conduct a communication well is to conduct it in a way that we would be comfortable with in times of vulnerability and fear. No one has to know many different societal customs, beliefs, or rules to provide exceptional care to people of any religion, ethnicity, or race. Perhaps, the secret of cultural competence is patient orientation. It demands respect, sensitivity, partnership, serenity, honesty, trust, curiosity, and tolerance. The most important interhuman need that unites all people is the desire to be cared for [13].

## Cultural awareness

Cultural awareness requires people to be aware of how their culture and that of others affect their behavior. Even if a person does not know much about other cultures, they should know about their own culture and how it shapes them. Being culturally aware can also mean to respect others’ cultures even if we do not understand every detail and every motivation affecting how to handle things. This is the starting point for intercultural understanding. The basis of an intercultural life is the awareness of cultural differences and how they affect the behavior of each individual. These influences affect both a person’s private and professional life [16]. However, intercultural awareness alone does not provide the ability to communicate across cultures. It is just the first step in a process.

As a continuation of this process in the healthcare field, Campinha-Bacote describes the continuous striving to perceive patients as a whole in their cultural context and to meet their needs. This includes the patient and their family [17]. This process requires cultural awareness and knowledge from everyone working in this field, as well as openness to cultural encounters and the ability to perceive cultural desires. In Campinha-Bacote’s work, cultural awareness is defined as a process in which, above all, one’s own prejudices against other cultures are examined. What is important here is the perception and illumination of one’s own cultural background. Some behaviors are perceived as inappropriate in daily dealings with foreign cultures. Cultural knowledge, as distinct from cultural awareness, is the process by which the physician learns the patient’s cultural history, particularly in dealing with illness and health, and cultural values and actions [17]. Leininger defined the concept of cultural evaluation. Here, a systematic assessment of individuals, groups, or communities in relation to their cultural values and practices is to be carried out in order to be able to determine the needs of these people [18].

## Intercultural communication

The roots of intercultural communication as a field of research lie in international economic relations, political contexts, and diplomatic exchanges. Currently, intercultural communication has become an important component of communication within a multicultural migration society. Considering high migration rates, everyone is very likely to get in intercultural contact during their everyday life. The first step toward intercultural exchange is the mastery of language. Worldwide, approximately 7139 different languages are spoken.

## Language skills

“If you talk to a man in a language he understands, that goes to his head. If you talk to him in his language, that goes to his heart.” *Nelson Mandela.*

Even well-integrated people fall into old learned behavior patterns in stressful situations like being in the hospital with a seriously ill child. This hampers communication about difficult issues with people from a different cultural background in a language they do not or hardly know. Even a person with good conversational skills in the language of the host country may not be able to fully understand, ask questions about, or read health-related information in that language. A California study from 2006 showed that the use of interpreters among Hispanic and Asian/Pacific Islanders (California’s two largest minorities) with poor language skills resulted in communication with office staff that was perceived as good and resulted in better access, more satisfying care, and a safer feeling in healthcare settings, compared to people from these groups who had good language skills but did not use interpreters. Hispanic and Asian/Pacific Islanders with limited language skills without interpreters reported significantly worse communication with office staff, more difficult access, less satisfying care, and an insecure feeling in healthcare settings. The authors concluded that the use of interpreters can help to deliver equally good medical care across language barriers for all ethnicities. Differential treatment based on race can thus be reduced [19]. For difficult conversations, a professional translator should always be consulted. Body language will still be observed even when an interpreter decodes the spoken language. Professional medical interpreters do not just translate the words; they preserve the sense and consonant meanings of the spoken text in translation. Medical translators must therefore have good medical knowledge and be familiar with the technical terms in both the source and target language.

During the COVID pandemic, we have come to appreciate the importance of telemedicine. Restrictions on numbers of hospital visitors make it difficult to allow several people to talk to a doctor, so that telemedical advice, e.g., via Zoom, is an alternative [20]. The importance of nonverbal aspects in a patient discussion must be considered here. Using telemedicine media, a possible loss of content of the conversation must be expected.

When a child whose health problem is the topic of a consultation in a pediatrician’s office, using the child as a translator should be avoided. Integrating children in institutions where they come into daily contact with the language and culture of the host country allows them to get to know the language and cultural nuances together with the children of the host country. Parents often rely on their children’s quickly learned skills. Parents expect their children to translate and interpret the new culture and language. It has been reported that such expectations and consequent obligations led to aggression, excessive risk-taking, and social problems in those children [21]. In 2019, Iranian colleagues analyzed the quality of the translation of doctor-patient interviews by non-professional translators. They found that the total amount of errors due to omitting or adding content when translating from the mother tongue into the official language were significantly more than the other way around, while mistranslation errors were almost the same. Another observation was that relatives with higher levels of education made fewer errors, and those living with patients made significantly more errors by adding content. These data are consistent with our own observations at our center. In summary, non-professional interpreters cannot effectively facilitate patient-physician communication, as their translation is error-prone, especially when translating from their native language into the official language [22, 23]. In patient care, it is important to understand exactly what needs to be translated. It is important for everyone involved that the translation enables an effective and secure communication between interlocutors. In written communication, it is important to remember that not everyone can read or write their own spoken language. This is particularly important for declarations of consent. Visualizing this content with images and diagrams can be helpful when a person lacks written language skills.

Forcibly displaced people form a special group within migrants. On the one hand, they suffer frequently from health problems after a long and arduous journey and therefore have to deal with the health system very soon and often after their arrival in the destination country. In addition, they have to be protected from further avoidable harm by paying special regard to their history. A lot of time is required in history-taking because trauma and shock may make it difficult to recall history correctly [24, 25].

## Intercultural communication skills

In intercultural communication, it is important to be aware of different communication styles in different cultures and how to interact with them. Disregarding cultural characteristics can lead to the patient receiving poor care, suffering damage, and being misdiagnosed. Schouten et al. [26] have analyzed intercultural medical communication difficulties and they found five key predictors of culture-related communication problems:

1. Cultural differences in perceptions of health and illness
2. Differences in cultural values
3. Cultural differences in desired doctor-patient relationships
4. Racism and prejudice
5. Language barriers

To counter these problems, it is important to acquire intercultural communication skills, such as listening, speaking clearly, and projecting positive body language. Those who understand how they communicate and how their culture shapes them can understand much more easily how they influence communication. It is important to know one’s own preferences, habits, and possible prejudices and stereotypes. Not before you have carefully perceived yourself should you focus on empathy with the interlocutor. Understanding that everybody has strongly been influenced by their own culture helps to create understanding for different cultural values. It also creates compassion, mindfulness, and empathy. To feel the needs of a patient in a communication and the role that is expected from oneself requires empathy. Empathy is an essential factor in intercultural communication. Mindful perception and empathy yield respect. Respect does not mean agreeing with a foreign culture in everything, but allowing others the right to express their values and culture. The high art of intercultural communication is reading between the lines, leading to “emotional intelligence.” This sort of intelligence requires intuition and the ability of non-verbal communication. It requires all senses and empathy to understand what is and is not being said. Intercultural communication requires a high degree of adaptability. We have to adjust our communication style to the conversational partner. Not only the way of speaking has to be adapted, we also have to listen and use body language, and have to be flexible in our thinking. Everybody needs to watch their own reaction to and engagement with other people. The most important skill for successful intercultural communication is perhaps patience as it moderates expectations and emotions. Paternotte et al. reviewed 145 publications dealing with the topic of intercultural communication. Linguistic, cultural, and social differences could be identified as difficulties in doctor-patient communication, as could mere assumptions and prejudices on the part of the doctors. Paternotte et al. concluded that the quality of intercultural communication could be improved by trainings for healthcare providers that highlight the issues of patient-centered communication, reciprocity, shared understanding, and shared decision-making [27].

## Anecdotal experiences of a Western pediatric nephrologist

If rules of communication are disrespected or communication skills are lacking, it will be hard to clearly communicate with patients from different cultures. It will cause a funny confusion at best, and, in the worst case, it will cause severe uncertainty and fear among parents and patients.

In our own experience, for example, a Western pediatric nephrologist who was known for his gentle nature did not consider the father of a patient to be very patriarchal regarding his culture and did not know that the mother never expects to be involved in the communication. When he approached the mother directly in good faith, she fainted because she was overwhelmed by the situation.

For a Western pediatrician, it is very important to involve the parents in the decision-making process, to be understanding and sensitive, and not to patronize patients. While attempting this, in some patients’ eyes, the doctor’s image may be insecure, inexperienced, and anxious. A listener from a different cultural background who expects directive communication to be appropriate in a situation may interpret participative communication as insecurity. If decisions are left to a patient who is used to very strong leadership, the patient will not be able to comply with this request; they will interpret the doctor’s behavior as hesitant and indecisive, and may assume that it is because the doctor does not believe in the effect of the drug him- or herself.

Sometimes, figurative examples are used to explain examinations more easily. Doing so, it might be easier to demonstrate the position you want your patient to take rather than explaining in words what they should do.

Just as important as knowing the culture of the person who is advised is to speak the language correctly or to work with a professional interpreter. The family we advised on the kidney transplant for their child claimed that they knew enough German to follow our clarification without an interpreter and that they would sign the consent. On the day of the transplant, we had to reassure an extraordinarily indignant father who stated that he would never have consented to a transplant if he had known that his child was getting a “used” kidney.

Also, the understanding of medical success may be completely different in different cultures and religion often plays an important role. In many Middle Eastern countries, consanguineous marriage is very common, often leading to the occurrence of recessive hereditary diseases. While many Western-oriented families would focus their entire lives on the stimulation and improvement of their child, in families from the Middle East, it could be the case that the success of the treatment and the survival of the child is placed in God’s hands. From the point of view of the treatment team, this often seems like non-adherence or neglect. From the parents’ point of view, it is the best way and safest thing they can do for their child. They are not neglecting the child just because they do not go to many therapies; rather, they are firm in their trust in spiritual help for the child. The conception of another child with the same disease can also be difficult to comprehend for non-Westerners. From their point of view, it may be the fulfillment of their duty imposed by God.

Although grief is something very personal, it can be experienced similarly in different cultures. In Western countries, it is not considered appropriate to cry and complain loudly, and the medical team often tries to keep their feelings to themselves. This can lead to situations in which doctors and nurses are perceived as not compassionate by grieving relatives and, conversely, the team perceives the relatives’ attitude as inappropriate in a hospital setting.

Of course, in a multicultural society, people from different backgrounds are getting closer and closer to each other, but if the expectations of the patient are completely different than those of the doctor, it is difficult to establish a trusting relationship.

Cultural behavior of patients and families different from that of those in the host country can lead to tensions in the relationship between the care team and the patient. A study from Italy showed that 28.5% of the nurses surveyed on their experiences in providing care to foreign children and their families did not feel sufficiently respected by parents from a different cultural background, and 16.1% said that they had experienced an overall disrespectful treatment of women during their work with these patients [28]. If one is aware of the role of women in many non-Western countries, one can approach this situation differently and it can help when processing these personally disappointing situations.

If doctors know that in many countries it is necessary that relatives of patients take on tasks in the hospital, such as transporting samples to the laboratory or bringing drugs needed from the pharmacy or being engaged in the supply of food, they may be less surprised when several adults accompany a child to the hospital. When caring for patients from a different culture, it is important to find a balance between both cultures—that of the home country and that of the host country. The WHO recommends recognizing patients as important, active players in improving their health. Patients must be fully supported in formulating their needs in order to make maximum use of the services they receive [29]. Patients from ethnic minorities need to be supported in finding a balance between preserving their culture of origin and what is possible in the cultural context of the hospital [16].

## Communication and religion in organ transplantation

It has been shown that while patients from minority ethnic communities have a higher risk of developing kidney failure, they are less likely to receive a kidney transplant [30]. Studies have shown that, in addition to cultural, social and educational issues, religious considerations, too, can play a role in deciding whether or not to have an organ transplant [31]. Migrants are likely to retain religious concerns in host countries [32]. In order to establish a productive relationship with patients and their families, it is important to be aware of one’s own knowledge gaps concerning the issues mentioned above.

When it comes to organ donation, religion and personal perception can contrast with each other. In 2010, Oliver et al. analyzed 10 religions, including Islam, Christianity, Judaism, Buddhism, and Taoism, and their attitudes toward organ donation. Interestingly, a clear ban on organ transplantation could not be found in any of the religions studied [33]. It is not the transplantation of an organ per se but the individual and emotional attitude to death and dying that can deter a person from organ donation. Nevertheless, a religion’s stand on death and dying must be considered carefully. Particularly important is the individual attitude of each patient toward their religion.

Obtaining consent for a transplant requires a strong awareness of religious concerns. A proactive approach to organ transplantation must always be balanced with the patient’s right to treat the issue of organ donation as a purely personal one [34]. Even if a member of the caregiver team belongs to the same religion as the patient, it is far from certain that they live and interpret the religion in the same way. The involvement of competent religious authorities in patient discussions can thus be helpful. On its website, the National Health Service (UK) offers the opportunity to find out about the attitude of different religions toward transplantation [35]. After all, fear of the unknown is an important contributor to communication difficulties.

## Conclusions

The way to good intercultural communication is via intercultural learning. Patients’ actions should be evaluated only after knowing the reasons for their behavior. Appreciation and respect for cultural differences and diversity, tolerance and respect for other people, empathy and adaptability, and critical self-reflection are skills that should be possessed by everyone who deals with people from other cultures. A trusting relationship between medical staff, patients, and their families does not necessarily require the ability to be fluent in every language, but the ability to listen to and observe people, if necessary with the help of an interpreter. Projecting the feeling that the other culture is respected will make it easier for patients and their families to feel safe in the healthcare network. We often expect people from another culture to adapt to new conditions and to embrace the prevailing values, opinions, and traditions of their new homeland. However, this is often not the case as people hold on to their own values and it is important that we understand this instead of judging. The first step in this process can even be recognizing the problem as a problem. This awareness will optimize care and communication in one’s own cultural bubble.

Sometimes it is beyond language and just requires humanity.
